# *In vitro* Quantification of Collagen and *Snail1* Gene Expression in Experimentally Induced Fibrosis by Arecoline and Commercial Smokeless Tobacco Products

**DOI:** 10.31557/APJCP.2020.21.4.1143

**Published:** 2020-04

**Authors:** Karunya Krishnakumar, Ramya Ramadoss, Rajkumar Krishnan, Hemani Sukhija

**Affiliations:** 1 *Apollo Specilaity Hospitals Chennai, *; 2 *Department of Oral Pathology and Microbiology, SRM Dental College, SRM University, Chennai, *; 3 *Index Institute of Dental Sciences Indore, India. *

**Keywords:** OSF, smokeless tobacco, arecoline

## Abstract

**Introduction:**

Extracellular matrix component derangement is the major event in pathogenesis of Oral submucous fibrosis. Many studies have elaborated the alteration of the matrix components at a cellular and genetic level. However elaborate quantification of the components with varying concentrations of Areca nut extract and commercial tobacco products have not been done so far.

**Materials and Methods:**

Primary culture of tissues sourced during crown lengthening procedures were used for establishment of fibroblast monoculture and fibroblast / keratinocyte co-culture. Extracts of areca nut, commercial smokeless tobacco products (gutkha and haans) and control CCl4 were tested at concentrations ranging from 20 μL, 40 μL, 80 μL, 160 μL, 320 μL and time intervals of 12, 24, 48, 72 hours. Collagen quantification by spectrophotometry and *SNAI1* gene expression study were done.

**Results:**

Extract of areca nut was found to show increased collagen production than commercial tobacco products and closely similar values to CCL4. Kruskal Wallis test was used to analyse the difference in collagen obtained. The mean values of collagen obtained in co-culture were lesser than those obtained in the fibroblast monoculture. *SNAI1 *gene expression was negative in both the culture experiments.

**Conclusion:**

Areca nut extract was found to be more potent as an individual agent. Commercial smokeless tobacco products Gutka and Hans exhibited increased collagen production at higher concentration. These findings further steps up the persuasive ill effects of tobacco products. Negative *SNAI1* gene expression was corroborated to lack of extracellular environment in the co coculture experiment.

## Introduction

Oral submucous fibrosis (OSF) is a chronic progressive potentially malignant disorder. OSF has a prevalence rate of 0.03% - 6.42% with high malignant transformation rate of 7.6% (Nair et al., 2014). Etiological factors which have been reported till date are chewing of Areca nut, capsaicin ; nutritional deficiency of vitamin A, B_12_, folate and Iron; defective Iron metabolism, high copper content, tobacco products, altered genetic and Immunologic Processes, accelerated cell mediated and humoral response (Sirsat et al., 1960; Ayinampudi et al., 2012; Khanna, 2006: Pindborg, 1972). Arecoline alkaloid present in Areca nut has been reported to be the chief etiological factor causing oral submucous fibrosis (Prabhu et al., 2014; Ahmad et al.,, 2006). 

Core pathogenetic event behind OSF is irreversible fibrosis (Wynn, 2009). Fibrosis occurring in OSF is due to defective collagen homeostasis. Decrease in collagen clearance is due to stabilization of collagen, defect in extracellular matrix (ECM) dynamics and inhibition of phagocytosis. Collagen type I is the predominant type present in the extracellular matrix of OSF (Ekanayaka and Tilakaratne, 2016). 

Genes play a vital role in determining the course of fibrosis. Numerous genes are either up-regulated or down regulated during the fibrotic disease process Pro-fibrotic genes propel the disease towards fibrosis and include growth factors like transforming growth factor- beta (TGFβ), and fibroblast growth factor (FGF2), collagen genes like collagen type I alpha 1 (COL1A1), collagen type I alpha 2 (COL1A2), and matrix metalloproteinases (MMP11, MMP12, MMP19 and MMP23) (Li et al., 2017). SNAI1 (Snail) is a zinc-finger transcription factor that belongs to a larger superfamily known as SNAI1 and participates in cell differentiation and survival. SNAI1 involves induction of epithelial mesenchymal transition (EMT) by suppression of E-cadherin transcription paving way for cell migratory capabilities, tumor progression, and metastases (Sugimachi et al., 2003). 

Morbidity and mortality associated with the disease process have instigated a series of research activities to unravel the mechanisms behind fibrosis. However treatment of oral submucous fibrosis have only been symptomatic till date and there is no specific treatment which gives a permanent reprieve from this debilitating disorder. Affected patients are left with minimal to moderate morbidity.

Many studies have elaborated the alteration of the matrix components at a cellular and genetic level, however elaborate quantification of the predominant component collagen with varying concentrations of Areca nut extract and commercial tobacco products in monoculture and co-culture with further validation of *SNAI1* gene expression have not been done so far. Stimulation of fibroblasts alone as a monoculture was thought of as it is the formative cell of collagen and fibroblasts are the key mediators of fibrosis in OSF. Conduct of a co-culture experiment with fibroblasts and keratinocytes is to allow interaction between two cell groups as they form the basic cellular schema of OSF.

## Materials and Methods

The study was approved by the Institutional Ethical Committee SRMDC/IRB/2016/MDS/NO.603.

*Source of tissue samples*

Tissues from third molar impaction surgery and periodontal surgical procedures were taken for establishment of fibroblast and keratinocyte cell line. 

Tissues from clinically and histopathologically diagnosed cases of OSF was taken for evaluation of *SNAI1* gene expression.

*Extract preparation*

Areca nut, Gutkha and Hans were grounded into coarse granules with help of mortar and pestle. 20 mg of the ground powder was mixed with 20ml of distilled water and boiled at 50^o^C for 20 minutes and then filtered using a Soxhlet apparatus.

*Cell culture studies*

*Establishment of primary fibroblast cell culture*

Tissues obtained were crushed into 1 mm × 1 mm × 1 mm pieces with help of tissue homogenizer and washed in Dulbecco’s phosphated buffer saline (DPBS). Tissue pieces were incubated for 18 h in working media “Dulbecco’s Modified Eagle Media augmented with 10% fetal bovine serum, 100 μg/ml of streptomycin, 1 μg/ml of amphotercin B and 100 μg/ml of penicillin comprising crude collagenase succeeding tissue were “centrifuged at 2,000 rpm for 5 min”. Media change was performed every 3^rd^ day. Confluency was reached in 2–3 weeks, after which the cells were subcultured. The cultures remained at “37°C in a humidified atmosphere of 95% air and 5% CO_2_”. The cells from fourth passage were used for the study. Morphologic characteristics were used to confirm the fibroblast lineage of the cultured cells.

*Establishment of primary keratinocyte cell culture*

Epithelium was expurgated into small bits using a sharp knife and TrypLE Express cell dissociation reagent was used to digest the epithelium. Keratinocytes obtained were sieved using a 100 micron Cell-Strainer, which was resuspended in 5 ml of medium consisting of low calcium and was shifted to a tissue culture incubator. 24 hours later non-adherent cells were removed by changing the medium. Low calcium containing medium was used to culture the keratinocytes, as they reach confluence subculturing using TrypLE was carried out. The cells from fourth passage were used for the study. 

*Monoculture establishment*

Individual culturing of isolated Fibroblasts was carried out using microwell tissue culture plates. 5,000 cells was inoculated in every well along with 100 µ1 of dulbecco’s modified minimal essential medium boosted with l-ascorbic acid (50 pg/ml), fetal bovine serum (l0%), sodium bicarbonate (0.37% w/v), streptomycin sulfate (100 ml), benzyl penicillin (200 ml), and l-glutamine (2 mm) (Sugimachi et al., 2003).

*Co-culture establishment*

Fibroblasts 0.15 or 0.3×10 were cultured in each 96-well culture- plates. To establish co-culture, 48 hours later, on top of the fibroblasts 1.0 or 1.5×10 keratinocytes were seeded. The culture medium used earlier for the commencement of fibroblast culture was the regular growth medium. From the time of initiation the medium used for co-culturing was changed periodically (Thiery et al., 2009).

*Induction of Experimental fibrosis*

After the establishment of mono and co-culture, cells in monolayer and co-culture were subjected to test drugs like arecoline Hydrobromide, gutkha, hans and carbon tetrachloride as control in succeeding concentrations 20 μL, 40 μL, 80 μL, 160 μL and 320 μL at time intervals of 12, 24, 48, 72 hours. 

*Quantification of collagen in monoculture and co-culture*

At 12, 24, 48 and 72 hours, from culture medium was aspirated from each well and the cells in the wells were fixed using methanol for 5 min. Cells were stained using 100μ1 methylene blue for 30 minutes and 400 μ1 borate buffer was used to rinse 3 times. 100 μ1 0.1 M HCl comprising 20% ethanol was used to elute methylene blue from the cell layers. Absorbance was measured using spectrophotometer at 650 nm.

*SNAI1 gene isolation*

SNAI1 gene isolation was done in OSF tissue samples and culture cells after induction of fibrosis. Total RNA was isolated using TRIZOL reagent. Reverse transcription was performed with 1 µg of total RNA and oligodeoxythymidylic acid primers. Quantitative PCR was performed using the SYBR green. *SNAI1* gene was amplified at (F: 5’-CGGGATCCTTCTTCTGCGCTACTGCTGCG-3’; R: 5’-CGGAATTCGGGCAGGTATGGAGAGGAAGA-3’). PCR conditions were kept at 2 min at 50°C and 10 min at 95°C for denaturation followed by 40 cycles consisting of 15 s at 95°C, and 1 min at 60°C. Target cDNA were observed as standard curves from which the rate of change of threshold cycle values were determined. 

## Results

*Collagen quantification in Fibroblast Monoculture*

*Quantification at different concentrations*

At a baseline concentration of 20 μl, the mean value of collagen obtained with arecoline Hydrobromide was higher than other experimental groups Gutka, Hans and control CCl_4_. Control group CCl_4_ exhibited the higher collagen secretion at 40 μl concentration. Hans exhibited higher values at 80 μl and 320 μl concentration and Gutka exhibited higher values at 160 μl concentration. The difference in mean rank values obtained at different concentration among the study groups were found to be statistically significant (Table 1).

*Quantification at different time periods*

Collagen production in fibroblast monoculture at 24, 48 and 72 hours resulted in a values which showed increased production by Conrol CCl_4_ than Arecoline Hydrobromide followed by Gutka and Hans. However, there was no significant statistical difference in the mean rank value among the groups (Table 2).

*Collagen quantification in Fibroblast/ Keratinocyte Co-culture*

*Quantification at different concentrations*

At baseline concentration of 20 μl the mean value of collagen obtained with Gutka and Hans were higher than arecoline Hydrobromide. Hans was higher than other experimental groups arecoline Hydrobromide at 40 μl. At concentration of 80 μl, 160 μl and 320 μl mean value of collagen obtained with arecoline Hydrobromide was higher than other experimental groups Gutka, Hans and control CCl_4_. The control group CCl_4_ exhibited the lowest value when compared to the experimental groups. The difference in mean rank values obtained at 80 μl ,160 μl and 320 μl concentration among the study groups was found to be statistically significant (Table 1).

*Quantification at different time periods*

Collagen production in Co-culture at 12, 24, 48 and 72 hours period resulted in values which showed increased production by Arecoline Hydrobromide than Gutka, Hans and control CCl_4_. However there was no significant statistical difference in the mean rank value among the groups (Table 2).

*Comparison of findings in monoculture and co-culture*

The mean values collagen obtained in co-culture was lesser than those obtained in the fibroblast monoculture. However the mean difference was not statistically significant difference between them.

*SNAI1 gene expression*

SNAI1 gene expression was done in OSF tissue samples and cultured cells induced of fibrosis. Tissue samples showed positive expression whereas both monoculture and coculture cells showed negative expression.

**Table 1:** Comparison of Collagen Secreted by Arecoline Hydrobromide, Gutkha, Hans and CCl4 in Monoculture and Co-culture in Different Concentrations - 20μl, 40μl, 80μl, 160μl, 320μl

		Monoculture			Co-Culture		
Concentrations	Drugs	Mean	P Value	Levels of Significance	Mean	P Value	Levels of Significance
20μl	Arecoline Hydrobromide	2.447500	0.005	Significant	0.767750	0.003	Significant
	Gutka	1.840000			0.781250		
	Hans	1.902500			0.789500		
	CCl_4_	2.665000			0.745500		
40μl	Arecoline Hydrobromide	2.570000	0.011	Significant	0.688750	0.003	Significant
	Gutka	1.962500			0.676750		
	Hans	2.705000			0.704500		
	CCl_4_	2.662500			0.568000		
80μl	Arecoline Hydrobromide	2.732500	0.005	Significant	0.631750	0.005	Significant
	Gutka	1.717500			0.579000		
	Hana	2.462500			0.575250		
	CCL4	2.672500			0.422500		
160μl	Arecolin Hydrobromide	2.555000	0.014	Significant	0.503500	0.005	Significant
	Gutka	2.627500			0.418500		
	Hans	2.345000			0.413500		
	Ccl4	2.660000			0.234250		
320μl	Arecoline Hydrobromide	2.767500	0.038	Significant	0.399500	0.003	Significant
	Gtka	2.880000			0.284250		
	Hans	2.955000			0.234000		
	CCl_4_	2.807500			0.131250		

**Table 2:** Comparison of Collagen Secreted by Arecoline Hydrobromide, Gutkha, Hans and CCl4 in Monoculture and Co-culture in Different Time Periods - 12, 24, 48 and 72 hours

	Drugs	Monoculture			Co-Culture		
		Mean	*P* Value	Levels of Significance	Mean	*P *Value	Levels of Significance
Time Periods							
12hrs	Arecoline Hydrobromide	2.522000	0.386	Not Significant	0.589800	0.645	Not Significant
	Gutka	2.124000			0.542000		
	Hans	2.396000			0.537400		
	CCl_4_	2.598000			0.414200		
24hrs	Arecoline Hydrobromide	2.596000	0.373	Not Significant	0.596000	0.645	Not Significant
	Gutka	2.178000			0.545400		
	Hans	2.450000			0.541000		
	CCl_4_	2.676000			0.417800		
48hrs	Arecoline Hydrobromide	2.640000	0.380	Not Significant	0.600800	0.690	Not Significant
	Gutka	2.224000			0.549800		
	Hans	2.498000			0.545400		
	CCl_4_	2.720000			0.422000		
72hrs	Arecoline Hydrobromide	2.700000	0.402	Not Significant	0.606400	0.690	Not Significant
	Gutka	2.296000			0.554600		
	Hans	2.552000			0.549600		
	CCl_4_	2.780000			0.427200		

**Figure 1:**
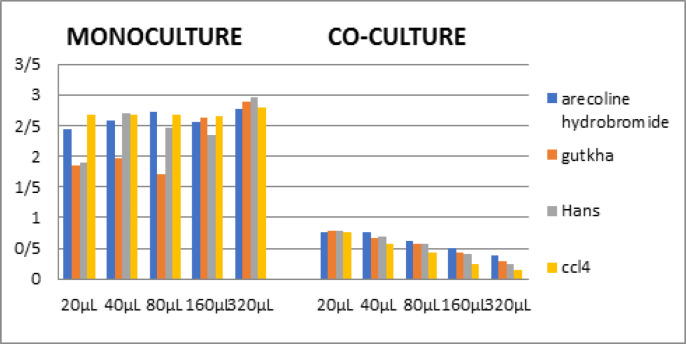
Comparison of Collagen Secreted by Arecoline Hydrobromide, Gutkha, Hans and CCl4 in Monoculture and Co-culture in Different Concentrations - 20μl, 40μl, 80μl, 160μl, 320μl

**Figure 2:**
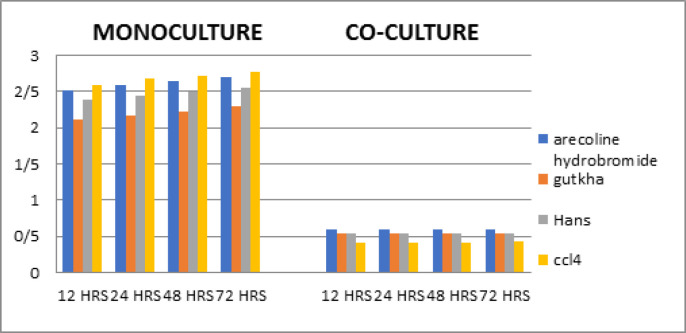
Comparison of Collagen Secreted by Arecoline Hydrobromide, Gutkha, Hans and CCl_4_ in Monoculture and Co-culture in Different Time Periods - 12, 24, 48 and 72 Hours

## Discussion

Deranged collagen metabolism is the key pathogenic event in chronic fibrotic disorders. Antifibrotic therapies have been shown to have reasonable success in management of chronic fibrotic disorders. However despite having a common paradigm, treatment for oral submucous fibrosis is predominantly symptomatic and not curative. Considering that the key role of the extracellular matrix, the predominant component of ECM -collagen was quantified by induction with known etiologic factors areca nut extract and commercial extracts. Quantification of collagen have been previously carried out with arecoline and arecaidine only. Commercial tobacco products with *SNAI1* gene expression were not done before. Profibrotic genes initiate the signaling cascade that up-regulates collagen production or down regulates collagen degradation, ultimately, driving a disease towards fibrosis. Analysing the gene expression of *SNAI1* will help delineate the fibrotic events.

Carbon tetrachloride was used as a control in the present study as its ability to induce experimental fibrosis was reported decades back.Collagen synthesis was also observed in-vitro from explant cultures of normal and CCl_4_ treated mouse liver (Korsud et al., 1973).

Fibroblast monoculture cell exhibited collagen production in increasing time periods and increasing concentrations Mean value of collagen obtained with arecoline Hydrobromide was higher than other experimental groups Gutka and Hans. The control group CCl_4_ exhibited the highest value when compared to the experimental groups. However the value of collagen produced by CCl_4_ was closely similar to arecoline hydrobromide. Though the individual roles of arecoline and CCl_4_ is well established in literature. There have been no study which compares the effects of both the chemicals in invitro culture. The values obtained in the study indicates that arecoline is as potent as CCl_4_. Considering the toxic carcinogenic effects of CCl_4_ and an equivalent behaviour by arecoline; it is an alarming finding as arecoline has widespread usage in various forms. However the percentage of arecoline in tobacco products varies and has to be thoroughly validated.

Co- culture experiments are done to study interactions between different types of cell populations. It forms the basis of many synthetic biology programs. Ample evidence-based literature suggests that progression of fibrotic events is vastly dependent on the bi-directional signaling exchanges between epithelium and mesenchyme. Conducting a co-culture experiment with fibroblasts and keratinocytes was considered as it would have a better purview in studying OSF (Wang et al., 2012). Fibroblast and keratinocyte co-culture experiment revealed that Arecoline was more potent as an individual agent, the concentration of which may vary in the commercial tobacco products Kumar et al., (2013). Yet, at higher concentrations the commercial products showed increased collagen production. This fact provides a compelling evidence on the potency of the tobacco products. Quantification of collagen production have not been done using commercial products Kumar et al., (2013). before. Quantification of collagen can be done by histochemical, imaging, photometric, microscopic methods. Present study used spectrophotometry as the method is easily accessible, cost effective, sensitive and specific. This study steps up the persuasive ill effects of tobacco products. 

Decreased collagen in co-culture in our study could be attributed to the fact that the presence of keratinocytes has altered the collagen secreting ability of fibroblasts. Nonetheless the pathogenic sequence of oral submucous fibrosis has substantial evidence in the reciprocal role of epithelial cells and the mesenchyme. Our study findings did not indicate the same. Reasoning out this finding, it is put forth that cell–cell interactions in co-culture experiment has a concerted role along with the extracellular environment. The present study can be expedited by conducting by replicating the environment in such a manner that the extracellular environment is also simulated close to an invivo environment which is a biggest challenge in conduct of co- culture experiment . 

The results showed values lesser than those obtained in the fibroblast monoculture experiment .Our results were similar with the findings by Xia et al., (2009) who reported decreased collagen with co-culture. They also had an additional finding that the Pro and active MMP-9 values increased in co-culture and was thought to be the reason behind decreased collagen in co-culture (Jeng et al., 1994).

Comparison of different time periods in co-culture was also evaluated. Collagen production in 12, 24, 48 and 72 hours period resulted in a values which showed increased production by Arecoline Hydrobromide than Gutka and Hans. Jeng et al., (1996) reported dose dependant reduction in collagen production with arecoline. However our study results differed with increased production with increased dose (Xia et al.,2009). 

Epithelial to mesenchymal transition (EMT) play a pivotal role in differentiation of tissues during embryogenesis. It also influences tissue repair and induce organ fibrosis and carcinoma progression. SNAI1 is has a crucial role in epithelial mesenchymal transition formation and maintenance of embryonic mesoderm, growth arrest, survival and cell migration (Lee et al., 2013).* SNAI1* gene expression was found to be positive in OSF tissues and negative in cultured cells induced of fibrosis. This finding was differed to the one conducted by by Lee et al., (2013) which showed that arecoline was found to elevate *SNAI1* expression in a dose-dependent manner both in human oral keratinocytes and oral cancer cell line. Our results in cultured cells did not exhibit *SNAI1* expression, this could be due to lack of an exact in-vivo environment. 

In summary, our experimental procedure was an humble attempt to mimic the pathogenic events in oral submucous fibrosis. The present study can be expedited by conducting by replicating the environment in such a manner that the extracellular environment is also simulated close to an in-vivo environment which is a biggest challenge in conduct of co- culture experiment. However, the study has effectual findings in proving the potency of Arecoline and the commercial tobacco products, Kumar et al., (2013). They have been similar to the proven carcinogen carbon tetrachloride which is a formidable evidence to be considered in creating blanket ban of smokeless tobacco products.
